# Hantavirus Pulmonary Syndrome Caused by Puumala Orthohantavirus—A Case Report and Literature Review

**DOI:** 10.3390/microorganisms11122963

**Published:** 2023-12-12

**Authors:** Marija Santini, Jelena Ljubić, Nikola Šoštar, Tatjana Vilibić-Čavlek, Maja Bogdanić, Samo Zakotnik, Tatjana Avšič-Županc, Miša Korva, Ivan Christian Kurolt, Leona Radmanić, Petra Šimičić, Juraj Krznarić, Branimir Gjurašin, Marko Kutleša, Klaudija Višković, Nataša Cetinić Balent, Renata Žunec, Ivana Margeta Marić, Ana Ribarović, Snjezana Židovec-Lepej

**Affiliations:** 1 Department for Infections in Immunocompromised Patients, University Hospital for Infectious Diseases “Dr. Fran Mihaljević”, 10000 Zagreb, Croatia; 2 School of Medicine, University of Zagreb, 10000 Zagreb, Croatia; tatjana.vilibic-cavlek@hzjz.hr (T.V.-Č.); jkrznaric@bfm.hr (J.K.); mkutlesa@bfm.hr (M.K.); 3 Infectious Diseases Department, County Hospital Čakovec, 40000 Čakovec, Croatia; jelljub@gmail.com; 4 Emergency Department, University Hospital for Infectious Diseases “Dr. Fran Mihaljević”, 10000 Zagreb, Croatia; nsostar@bfm.hr; 5 Department of Virology, Croatian Institute of Public Health, 10000 Zagreb, Croatia; maja.bogdanic@hzjz.hr; 6 Institute of Microbiology and Immunology, Faculty of Medicine, University of Ljubljana, 1000 Ljubljana, Slovenia; samo.zakotnik@mf.uni-lj.si (S.Z.); tatjana.avsic@mf.uni-lj.si (T.A.-Ž.); misa.korva@mf.uni-lj.si (M.K.); 7 Research Department, University Hospital for Infectious Diseases “Dr. Fran Mihaljević”, 10000 Zagreb, Croatia; ikurolt@bfm.hr; 8 Department for Molecular Diagnostics and Flow Cytometry, University Hospital for Infectious Diseases “Dr. Fran Mihaljević”, 10000 Zagreb, Croatia; leona.radmanic@gmail.com (L.R.); petrasimicic@gmail.com (P.Š.); szidovec@gmail.com (S.Ž.-L.); 9 Department of Intensive Care Medicine and Neuroinfectology, University Hospital for Infectious Diseases “Dr. Fran Mihaljević”, 10000 Zagreb, Croatia; bgjurasin@bfm.hr; 10 Department of Radiology and Ultrasound, University Hospital for Infectious Diseases “Dr. Fran Mihaljević”, 10000 Zagreb, Croatia; kviskovic@bfm.hr; 11 Department of Clinical Microbiology, University Hospital for Infectious Diseases “Dr. Fran Mihaljević”, 10000 Zagreb, Croatia; ncetinic@bfm.hr; 12 Tissue Typing Laboratory, University Hospital Zagreb, 10000 Zagreb, Croatia; renata.zunec@kbc-zagreb.hr; 13 County Hospital “Dr. Josip Benčević”, 35000 Slavonski Brod, Croatia; ivana2mar@gmail.com; 14 County Hospital Zadar, 23000 Zadar, Croatia; anaribarovic@net.hr

**Keywords:** hantavirus, hantavirus pulmonary syndrome, acute respiratory distress syndrome, Puumala orthohantavirus, immunomodulatory therapy, chemokine profile

## Abstract

In this article, we report on a rare case of acute respiratory distress syndrome (ARDS) caused by the Puumala orthohantavirus (PUUV), which is typically associated with hemorrhagic fever with renal syndrome (HFRS). This is the first documented case of PUUV-associated ARDS in Southeast Europe. The diagnosis was confirmed by serum RT-PCR and serology and corroborated by phylogenetic analysis and chemokine profiling. The patient was a 23-year-old male from Zagreb, Croatia, who had recently traveled throughout Europe. He presented with fever, headache, abdominal pain, and sudden onset of ARDS. Treatment involved high-flow nasal cannula oxygen therapy and glucocorticoids, which resulted in a full recovery. A systematic literature review identified 10 cases of hantavirus pulmonary syndrome (HPS) caused by PUUV in various European countries and Turkey between 2002 and 2023. The median age of patients was 53 years (range 24–73), and six of the patients were male. Most patients were treated in intensive care units, but none received antiviral therapy targeting PUUV. Eight patients survived hospitalization. The presented case highlights the importance of considering HPS in the differential diagnosis of ARDS, even in areas where HFRS is the dominant form of hantavirus infection.

## 1. Introduction

Hantaviruses are a diverse group of rodent-borne zoonotic viruses that belong to the genus *Orthoantavirus* of the family *Hantaviridae*. In humans, hantaviruses cause two distinct diseases: hemorrhagic fever with renal syndrome (HFRS) and hantavirus pulmonary syndrome (HPS). HFRS is caused by Old World orthohantaviruses Puumala (PUUV), Dobrava (DOBV), Seoul (SEOV), and Hantaan (HTNV) in Europe and Asia. New World orthohantaviruses cause HPS, which is endemic in the Americas. Although HFRS and HPS are distinct clinical entities, they share several features and overlapping symptoms [1]. The clinical presentation of HFRS ranges from asymptomatic or mild to severe, occasionally fatal infection, depending in part on the causative virus. PUUV and SEOV generally cause a mild form of the disease with a mortality rate of less than 1%. In contrast, DOBV and HTNV cause more severe clinical forms that result in fatality rates between 5% and 15% [2]. HPS is an even more severe disease characterized by respiratory failure with diffuse interstitial edema and a mortality rate of 38% [3].

After the first description of HFRS in Croatia in 1952 [4], outbreaks and sporadic infections are continuously reported. The first large outbreak, which included 125 cases, was recorded in 1995 in several well-known endemic foci (Gorski Kotar, Lika, Slunj, Mala Kapela, and Slavonia) [5]. The following outbreaks in 2002 (401 cases) [6,7], 2012 (154 cases), 2014 (209 cases) [8], 2017 (389 cases), 2019 (197 cases), and 2021 (334 cases) [9] indicated that all of Croatia except for the coastal region and the islands is endemic for hantavirus infections [8,9,10]. PUUV is the most commonly detected orthohantavirus in Croatia (80–90%), while DOBV infections were also recorded. PUUV-infected patients mainly presented with fever (100%), headache (88.3%), backache (87.2%), oliguria (56.4%), and anuria (7.5%). Hemorrhagic manifestations were more frequently observed in DOBV-infected patients [11,12].

HFRS can be accompanied by the signs and symptoms of respiratory system involvement, but usually in a mild form. So far, only individual cases and one case series of HPS caused by PUUV have been published. We present a case of HPS detected in Croatia during the 2022 outbreak. This is the first documented case of HPS caused by PUUV in Southeast Europe, supported by virus sequencing, chemokine profile, and a review of the cases published so far.

## 2. Materials and Methods

### 2.1. Serological Methods

Rapid diagnostics of acute hantavirus infection was performed using serum samples on ReaScan+ PUUMALA IgM and ReaScan DOBRAVA-HANTAAN IgM (Reagena, Toivola, Finland) immunochromatographic assays (ICAs). Additional serology was done using indirect immunofluorescence assay (IFA), Hantavirus mosaic (Euroimmun, Lübeck, Germany) to detect IgM and IgG antibodies for the most common orthohantaviruses: PUUV, DOBV, HTNV, SEOV, Saaremaa, and Sin Nombre. A positive result was fluorescence appearing as fine droplets in the infected cells’ cytoplasm in a dilution of 1:100.

### 2.2. RT-PCR and Next-Generation Sequencing

Viral nucleic acid was isolated from 400 µL of serum using the Viral RNA Extraction kit on a BioMagPure 12 Plus (Biosan, Riga, Latvia). In a nested RT-PCR system using PUUV-specific primers, described elsewhere [13,14], a 341 bp fragment of the nucleocapsid protein was amplified.

#### 2.2.1. Sample Preparation for Next-Generation Sequencing

RNA was isolated from the serum sample using the EZ1 Virus Mini Kit v2.0 on an EZ1 Advanced XL instrument (Qiagen, Hilden, Germany) according to the manufacturer’s instructions. The isolated nucleic acid was treated with the Turbo DNA-free Kit to remove DNA according to the manufacturer’s instructions for rigorous DNA removal. The purified nucleic acid was used for cDNA synthesis according to the SISPA protocol [15]. Briefly, we synthesized double-stranded cDNA using the Maxima H Minus Double-Stranded cDNA Synthesis Kit (Thermo Scientific, Waltham, MA, USA), but instead of random hexamers, we used a SISPA-A primer for the synthesis of the first strand of cDNA. The second strand was synthesized according to the protocol of the kit. The double-stranded cDNA was cleaned with a GeneJET PCR Purification Kit (Thermo Scientific). Next, we used 10 µL of double-stranded cDNA as a template for the SISPA-B reaction using a SISPA-B primer for cDNA amplification according to SISPA protocol [15]. Amplified cDNA was cleaned with AMPure XP beads (Beckman Coulter) at a 1:1 ratio. We measured the final concentration of double-stranded cDNA after the first SISPA-A and second SISPA-B reactions using the Qubit 3.0 and High Sensitivity dsDNA Assay Kit (both Thermo Scientific) and fragment size with the Bioanalyzer 2100 using the Agilent High Sensitivity DNA Kit (both Agilent).

#### 2.2.2. Next-Generation Sequencing Library Preparation and Sequencing

Because we had synthesized sufficient cDNA using the first SISPA-A and SISPA-B reactions, we used both for NGS library preparation. We prepared NGS libraries using the Nextera XT kit (Illumina, San Diego, CA, USA) in combination with IDT for Illumina UD Indexes set C (Illumina) according to the manufacturer’s instructions. NGS libraries were sequenced on NextSeq 550 (Illumina) in a 149 bp long pair-end configuration using NextSeq 500/550 High Output Kit v2.5 (300 cycles).

#### 2.2.3. Bioinformatic Analysis

The generated reads were checked for quality using FastQC (v. 0.11.5) [16]. Adapters and low-quality reads were trimmed using BBduk (v. 38.98) [17]. Trimmed reads were used for de novo assembly using MetaSPAdes (3.15.4) [18] with options -k 21,33,55,77,99,127. The generated contigs were translated into amino acids and aligned to the NCBI NR database (base downloaded April 2021) using DIAMOND (v. 2.0.14.152) with default settings [19]. Taxonomic analysis was performed using Megan 6 (v. 6.22.2) [20]. All contigs aligned to PUUV were confirmed with the online BLAST tool. Phylogenetic analysis was performed with IQTree (v. 2.2.0 COVID-edition) [21,22,23,24,25] using the TIM2 + F + G4 model. The phylogeny tree was constructed using TVBOT (v. 2.6) [25].

### 2.3. Chemokine Determination

Concentrations of chemokines including CCL2, CXCL10, CCL11, CCL17, CCL3, CCL4, CXCL9, CCL20, CXCL5, CXCL1, CXCL11, and IL-8 (CXCL8) in the plasma were determined by multiplex-based flow cytometry assay (LEGENDplex Proinflammatory Chemokine Panel 1, BioLegend, San Diego, CA, USA) on a FACS Canto II instrument (Beckton Dickinson, Franklin Lakes, NJ, USA).

### 2.4. Literature Search for Hemorrhagic Fever with Pulmonary Syndrome

A literature search was conducted in the electronic databases PubMed, Web of Science, Medline, and Scopus using the keywords “Puumala virus” OR “Puumala” AND “pulmonary” OR “pulmonary syndrome”. Only articles involving adult patients diagnosed with pulmonary syndrome and PUUV infection proven by serological or molecular methods in European countries published in the English language were selected. Only studies providing detailed information on clinical presentation, treatment, and short-term outcomes were eligible. The searches were performed up to May 2023. The reference lists of all included studies were also screened. Articles published up to May 2023 were included. A list of previously published cases and case series was created. The data on demographic characteristics, clinical presentation, treatment, length of intensive care unit, hospital stay, and vital status at discharge were extracted. Publication searches and data extraction were performed by two researchers (J.L. and N.Š.) under the supervision of M.S.

## 3. Results

### 3.1. Case Report

On 30 October 2022, a 23-year-old male presented to the University Hospital for Infectious Diseases, Zagreb, Croatia, due to a four-day fever (max. 40.0 °C), headache, nausea, and upper abdominal pain without respiratory symptoms. At home, he was taking acetaminophen. Due to chronic urticaria, diagnosed two years before, he was treated with desloratadine. The patient was a non-smoker. He was a sporadic case, residing in Zagreb but traveling abroad. At the beginning of September, he was in Malmö, Kullaberg Nature Reserve (Sweden), and Copenhagen (Denmark). By mid-September, he visited Plovdiv, Sofia (Bulgaria), and Milan (Italy) by mid-October. He trekked on the Medvednica mountain near Zagreb from mid-September to mid-October. The patient was a man having sex with a man (MSM) with one new partner during the last eight weeks. The patient denied evident vector exposure. He was vaccinated according to the national immunization program, including three mRNA vaccine doses for SARS-CoV-2. A rash upon receiving amoxicillin-clavulanic acid was reported. 

**Figure 1:**
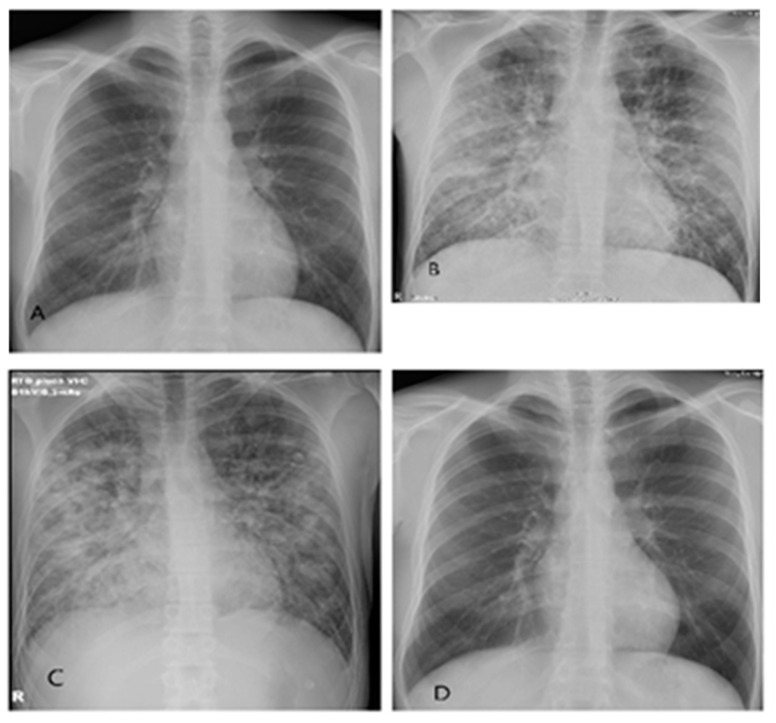
Series of thoracic X-rays in a patient with HPS caused by PUUV: (**A**) 30 October 2022, normal appearance of lungs and heart at admission. (**B**) 31 October 2022 (6 h interval), bilateral diffuse interstitial and alveolar infiltrates in all pulmonary fields. (**C**) 3 November 2022, very good regression of bilateral diffuse interstitial and alveolar infiltrates. Small bilateral basal pleural effusions. (**D**) 10 November 2022, complete regression of bilateral diffuse interstitial and alveolar infiltrates and pleural effusions.

At the first exam, the tympanic temperature was 37.7 °C, blood pressure 128/60 mmHg, heart rate 100/min, respiratory rate 18/min, and SpO_2_ 98% on room air. Other findings were unremarkable. The results of the laboratory tests and the initial chest X-ray are presented in Table 1 and Figure 1A. At the time of arrival, moderately elevated values of C-reactive protein (CRP) and procalcitonin, thrombocytopenia, high values of fibrinogen and D-dimer, hypoproteinemia, hypoalbuminemia, increased urine specific gravity, and positive proteins in urine were observed. The results of the other laboratory tests were unremarkable. The electrocardiogram (ECG) demonstrated sinus rhythm, 100 beats per minute, with other unremarkable ECG elements. After the six-hour emergency department observation, the patient reported shortness of breath, and desaturation was recorded (SpO_2_ 80% on room air) with inspiratory crackles over both lungs. The patient was admitted to the intensive care unit. PaO_2_/FiO_2_ was 123 mm Hg, demonstrating moderate acute respiratory distress syndrome (ARDS). The patient was put on high-flow nasal cannula (HFNC) oxygen, 60 L/min, FiO_2_ 100%. The second lung X-ray is shown in Figure 1B. At the admission, the patient had a normal estimated glomerular filtration rate of 110 mL/min/ 1.73 m^2^ (eGFR, Table 1) which was calculated using the 2021 Chronic Kidney Disease Epidemiology Collaboration Creatinine equation (CKD-EPI) [26]. Abdominal and chest ultrasound showed marginal hepatomegaly, splenomegaly (14 cm), enlarged kidneys, and pleural effusion (12 mm) in the left hemithorax base. The transthoracic echocardiogram was unremarkable. Ceftriaxone 2 g q24h IV, azithromycin 500 mg q24h IV, and oseltamivir 75 mg q12h PO were started. 

Antimicrobial therapy was discontinued on the second in-hospital day after getting positive results of serology and RT-PCR for PUUV in serum and negative bacteriology. Methylprednisolone 250 mg q24h IV with rapid tapering was administered for six days. A gradual recovery of hypoxemia was observed. The patient was supplemented with oxygen by HFNC for three days and one more day by nasal catheter. Radiological and laboratory improvement was also noted, as seen in Figure 1C and Table 1. There was a decrease in the CRP level, mild anemia, and an increase in the platelet number.

On the 10th day of illness, the patient complained of bilateral lumbar discomfort. Decreased eGFR (52 mL/min/1.73 m^2^) and polyuria were observed. On the 12th day, a fourfold increase in aminotransferases was recorded, with hypoproteinemia and hypoalbuminemia but with normal urine specific gravity and lower proteinuria (Table 2). Tests for viral hepatitis were negative. The patient’s general condition was remarkably improved. 

The patient was discharged home on the 16th day since disease onset, in good condition but still with decreased eGFR (73 mL/min/1.73 m^2^). At the follow-up, four weeks after admission, the patient complained of bilateral lumbar discomfort. His eGFR (129 mL/min/1.73 m^2^) was normal, as were his other laboratory results. The chest X-ray was unremarkable (Figure 1D). At the follow-up, 10 weeks after admission, he was completely recovered.

### 3.2. Microbiology Results

The initial microbiology evaluation demonstrated negative nasopharyngeal swab multiplex PCR/RT-PCR for influenza A and B, RSV, SARS-CoV-2, adenoviruses, bocavirus, enteroviruses, human metapneumovirus, other coronaviruses, parechoviruses, rhinoviruses, and parainfluenza viruses type 1–4. Legionella urine antigen test was negative. Blood cultures and urine cultures were sterile. Rapid ICA demonstrated positive IgM for PUUV while negative for DOBV. The patient’s serum was positive for PUUV by RT-PCR. The patient was serologically negative for HIV-1/2 with undetectable HIV-1 RNA in the plasma. 

**Figure 2:**
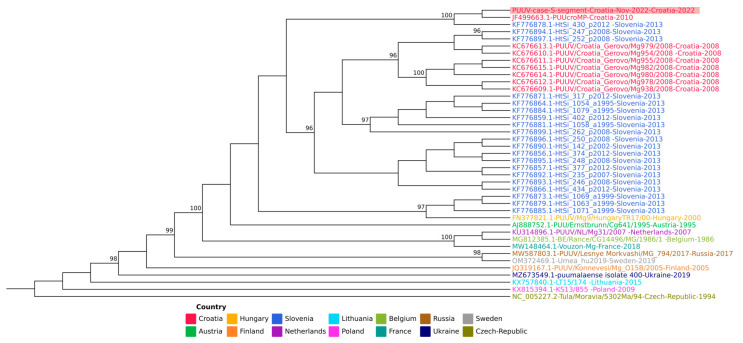
A subset of 40 PUUV S segments, emphasizing the geographic closeness of the origin countries is presented. Sequences are color-coded by the country of origin. A new sequence of the PUUV S segment from Croatia (marked with a red background) clusters with existing Croatian sequences. The value on the branches represents an ultrafast bootstrap approximation (only values above 95 are shown). The sequence of the Tula virus was used as a root (NC_005227.2).

The result of phylogenetic analysis is demonstrated in Figure 2. After trimming, we had 51,075,339 pair-end reads from SISPA A and 239,689,976 pair-end reads from SISPA B. Using de novo assembly, we generated a nearly complete S-segment of PUUV with a length of 1657 bp. We aligned the obtained PUUV S segment sequence with PUUV S sequences deposited to NCBI from Croatia and neighboring European countries using MAFFT (v. 7.505) [27]. 

### 3.3. Chemokine Concentrations and HLA Typing

The chemokine profiles of the patient are demonstrated in Table 2. On 3 November and 7 November 2022, there were increased concentrations of CXCL9 (3690.8 and 3055.6 pg/mL, respectively) and CCL2 (778.8 and 462.6 pg/mL), above the range previously reported for normal human plasma (range 8–491 pg/mL for CCL9 and 37–351 pg/mL for CCL2).

The patient’s HLA alleles were tested by PCR SSO/SSP. He was HLA-A 24 66, HLA–B 13 41, HLA–C 06 17, HLA-DRB1 07-, and HLA-DQB1 02 03 positive. 

### 3.4. Literature Review

After the literature search, we documented seven articles from which data regarding demographics, clinical presentation, treatment, and treatment outcomes could be extracted. The results are presented in Table 3 and Table 4. There were two articles each from Sweden and Finland and one each from Italy, Belgium, Germany, Turkey, and Spain. The papers were published between 2002 and 2020. These are individual case reports or case series of up to three patients for a total of 10 patients, with a median age of 53 (range 24–73) years old. There were six male patients. The most common signs and symptoms were fever, upper abdominal pain, dyspnea, and cough. The median duration of the disease at admission was three days (minimum two, maximum four days). All patients, except one, were treated in the intensive care unit (ICU). The median ICU length of stay (LOS) was 13 (range 1–39) days, while the median hospital LOS was 15.5 (range 7–55) days. Eight of ten patients survived hospitalization. Mechanical ventilation was used in six, hybrid veno-arterial (VA) and veno-venous extracorporeal membrane oxygenation (VV ECMO) in one, and continuous renal replacement treatment (CRRT) in five patients. Eight patients were on vasopressors. Glucocorticoids were used in five patients, antibiotics in eight, and antivirals empirically (acyclovir and oseltamivir) in two patients, but with no attempts for targeted treatment of hantaviral infection. 

## 4. Discussion

This article presents a patient with a PUUV infection confirmed by serology and RT-PCR, dominantly manifested by abrupt ARDS development. Nevertheless, renal injury, a hallmark of PUUV infection, was mild and transient.

A thorough literature review revealed ten adult patients with PUUV infection and ARDS, defined as HPS [28,29,30,31,32,33,34] and described as case reports and short case series originating from different European countries during 18 years. All the patients initially had non-specific infectious disease symptoms for two to three days, followed by sudden respiratory deterioration. PUUV was diagnosed in the diagnostic workup for renal injury. Most patients were given antibiotics and some influenza antivirals, probably unnecessarily, due to suspected bacterial coinfection or suspected severe influenza in the circumstances of abrupt respiratory failure. There were no attempts to target PUUV. Half the patients received glucocorticoids, whose position in treating hantavirus infections is still controversial. However, despite the severe form of the disease, most patients survived by receiving intensive treatment. Our patient’s clinical characteristics were like those of the patients described so far. 

The PUUV HPS case and the literature review we present highlight several discussion points. PUUV is not commonly identified as a cause of ARDS or HPS, possibly because clinicians do not consider it a potential causative agent of acute hypoxemic respiratory failure. The case we presented was particularly diagnostically challenging, as it occurred during mid-autumn when multiple respiratory viruses were circulating, influenza virus was on the rise, and hantavirus infections were infrequent. However, after detecting thrombocytopenia and proteinuria and considering the patient’s frequent outdoor activities and travel history, diagnostic tests confirmed a PUUV infection acquired locally. So, this is the first documented case of HPS caused by PUUV in Southeastern Europe. 

Phylogenetic analysis of the obtained viral sequence showed a 97% identity to the sequence from a previous patient identified in 2010 in the area surrounding Zagreb, suggesting a locally acquired infection during leisure activities in this important recreational area. These two sequences, obtained from patients from our hospital, separate on the phylogenetic tree from a cluster of sequences obtained from rodents trapped in the Croatian region of Gorski Kotar [35], as well as from sequences obtained in Slovenia. This fact demonstrates the wide genetic diversity of PUUV.

Extensive immune activation and the development of a “cytokine storm” play an important role in the pathogenesis of severe hantavirus infections. Analysis of cytokine and chemokine expression in HPS and HFRS showed distinct biomarker profiles. HPS is associated with upregulating innate immunity cytokines and Th1-type responses [36]. The chemokine profile of the patient described in this article included high expression of CCL2 and CXCL9. CCL2 is one of the main regulators of monocyte and macrophage recruitment to the sites of inflammation that also promotes tumor dissemination, invasion, and immune evasion [37]. CXCL9 is an IFN-γ-inducible chemokine that regulates T-cell migration, differentiation, and activation [38]. Both chemokines are involved in the pathogenesis of various infectious and malignant diseases [37,38]. The high concentration of CCL2 and CXCL9 detected in our patient is in accordance with the chemokine profiles of both HPS and HFRS patients described by Khaiboullina et al. (2017) [36]. Therefore, our results suggest that chemokines regulating monocyte/macrophage and T-cell migration to sites of infection play an important role in the pathogenesis of severe hantavirus infection. In addition, a marked reduction in the CCL2 concentration observed on day 12 of the disease in our patient coincided with the significant improvement of the patient’s clinical condition.

In a 2019 study by Garanina et al., it was shown that patients with fatal HFRS caused by PUUV had increased concentrations of CCL5, IL-18, stem cell growth factor-β, and tumor necrosis factor-β [39]. Additionally, a study conducted by Korva et al. (2019) demonstrated overexpression of CCL3, IL-6, and interferon-α2 in fatal HFRS, indicating its significant pathogenic contribution in severe cases [40]. 

It is known that acute kidney injury requiring dialysis during HFRS is more likely to occur in individuals with human leukocyte antigen (HLA) B8 and DR3 alleles [41], while those with HLA B27 tend to have milder disease [42]. However, our patient’s HLA genotyping did not match up with these HLA alleles. 

The patient presented in this report had chronic urticaria, which could indicate immune dysregulation. However, it is unclear if this condition directly affected the immune response to hantavirus infection. Further research and investigation are needed to determine its specific impact on the response to hantavirus or other infectious agents.

Treating the hantavirus infection presented a therapeutic challenge because there is currently no clinical guidance. There are no specific antiviral drugs for hantaviruses, though one study showed that intravenous ribavirin reduced mortality [43]. However, subsequent studies did not confirm this. The effectiveness of glucocorticoids is also inconsistent [44]. In some cases, patients with severe PUUV infection were treated with icatibant, a drug used to treat acute hereditary edema [45,46]. Without clear guidance, we decided to administer short-term immunomodulatory treatment with methylprednisolone. The patient responded favorably.

This study has some limitations that need to be addressed. It is based on only one case, which restricts the generalization of the findings. Additionally, the number of reviewed cases is small, possibly due to publication bias, as cases with a positive clinical outcome are more likely to be published, and the study only considered cases published in English-language journals.

This case and literature review offer valuable insights into a distinct type of PUUV infection. They provide detailed information on patients’ clinical presentation, diagnosis (including phylogenetic analysis), treatment, and an in-depth analysis of cases published from 2002 to the present day.

## 5. Conclusions

The presented case study highlights an unusual presentation of PUUV infection, emphasizing the significance of considering PUUV as a cause of ARDS in clinical practice. While PUUV infections are frequently associated with HFRS, this case highlights the diverse clinical spectrum of this zoonotic virus.

Analyzing the immune response, elevated concentrations of specific chemokines (CCL2 and CXCL9) were detected, suggesting their role in the immunopathogenesis of severe hantavirus infections. These findings warrant further research into the immune response and potential immunomodulatory interventions for hantavirus-associated diseases.

This article discusses the difficulties in treating severe hantavirus infections. There is a lack of evidence regarding the effectiveness of antiviral drugs and controversy surrounding using glucocorticoids for immunomodulation. Treatment mainly involves supportive measures in intensive care units. However, prompt recognition and intensive treatment can be lifesaving.

In summary, this study contributes to increasing knowledge on hantavirus infections, emphasizing the diversity of clinical presentations and the importance of vigilance, even in regions with limited previous experience with HPS.

## Figures and Tables

**Table 1:** Main laboratory findings of the patient with HPS caused by PUUV.

Parameter	31 October 2022	3 November 2022	7 November 2022	Reference Range
C-reactive protein, mg/L	182.3		10.5	<5.0
Procalcitonin, µg/L	0.761			<0.5
Interleukin-6, ng/L	199.5			<7.0
Lactate, mmol/L	1.25			1.10–2.20
White blood cells, ×10^9^/L	6.9	15.6	14.6	3.4–9.7
Segmented neutrophils, %	63.7	68.6	71.4	44–72
Lymphocytes, %	15.8	18.5	15.0	20–46
Monocytes, %	13.9	12.3	11.2	2–12
Eosinophils, %	5.8	0.1	1.6	0–7
Basophils, %	0.8	0.5	0.8	0–1
Red blood cells, ×10^12^/L	4.81	4.30	3.81	4.34–5.72
Hemoglobin, g/L	148	131	116	138–175
Hematocrit, g/L	0.438	0.382	0.341	>0.415
Thrombocytes, ×10^9^/L	23	72	338	158–424
Serum sodium, mmol/L	140	140	145	137–146
Serum potassium, mmol/L	4.0	4.5	4.3	3.9–5.1
Serum chloride, mmol/L	103	104	108	97–108
Serum glucose, mmol/L	5.6		4.9	4.2–6.0
Serum urea, mmol/L	5.7	8.4	9.3	2.8–8.3
Serum creatinine, µmol/L	87	90	133	64–104
Total bilirubin, µmol/L	18			3–20
Aspartate aminotransferase, U/L	27		56	11–38
Alanine aminotransferase, U/L	13		168	12–48
Gamma-glutamyl transpeptidase, U/L	15		117	11–55
Alkaline phosphatase, U/L	44		85	60–142
Lactate dehydrogenase, U/L	195		161	<241
Creatine kinase	51		26	<177
Hs-Troponin T, µg/L	0.011			<0.1
NT-pro-BNP, ng/L	200			0–100
Serum total protein, g/L	57		56	66–81
Serum albumin, g/L	33.6		30.8	40.6–51.4
Prothrombin time ratio	0.86		1.12	>0.70
International normalized ratio, INR	1.10		0.98	2.0–3.0 for anticoagulation
Fibrinogen, g/L	4.4			1.8–3.5
D dimers, mg/L	1.69			<0.55
Urine pH	5.0		5.5	5.0–9.0
Urine-specific gravity, kg/L	1.041		1.007	1.005–1.030
Urine-nitrates, negative or positive	negative		negative	negative
Urine-leukocyte esterase, negative to 3+	1+		1+	negative
Urine-erythrocytes, negative to 4+	1+		1+	negative
Urine-ketones, negative to 4+	1+		negative	negative
Urine-proteins, negative to 4+	3+		1+	negative
Urine sediment-white cells (N/v.f.×400)	1–2		1–2	0–2

**Table 2:** Levels of serum chemokines on the eighth and twelfth day from the disease onset in a patient with HPS caused by PUUV.

Parameter	3 November 2022	7 November 2022	Controls (Range) **
IL-8, pg/mL	61.6	20.2	not detected—2704
CXCL10, pg/mL	2474.9	1169.3	13–1700
CCL11, pg/mL	49.8	204.1	not detected—18,866
CCL17, pg/mL	11.4	264.3	3–253
CCL2, pg/mL	778.9	462.6	37–351
CCL3, pg/mL	264.9	310.1	not detected—924
CXCL9, pg/mL	3690.8	3055.6	8–491
CXCL5, pg/mL	<12.6	227.9	6–326
CCL20, pg/mL	10.96	16.8	not detected—193
CXCL1, pg/mL	27.5	100.8	not detected—359
CXCL11, pg/mL	782.7	536.8	not detected—1356
CCL4, pg/mL	13.4	5.0	not detected—57

Interleukin-8 (IL-8); interferon-gamma induced protein 10 (CXCL10); eotaxin (CXCL11); thymus- and activation-regulated chemokine (CCL17); monocyte chemoattractant protein-1 (CCL2); macrophage inflammatory protein-1 alpha (CCL3); monokine induced by gamma interferon (CXCL9); epithelial neutrophil-activating peptide 78 (CCL5); macrophage inflammatory protein-3 alpha (CCL20); growth-regulated alpha protein (CCL1); interferon-inducible T-cell alpha chemoattractant (CXCL11); macrophage inflammatory protein-1 beta (CCL4). ** Chemokine concentrations in 60 normal human plasma samples (based on LEGENDplex Multi-Analyte Flow Assay Kit, Human proinflammatory chemokine panel 1 manual. Available at https://www.biolegend.com/nl-be/products/legendplex-hu-proinflam-chemokine-panel-1–13-plex-wvbp-20160, accessed on 17 July 2023).

**Table 3:** Review of cases and case series of HPS caused by PUUV published so far—main clinical features and treatment outcomes.

Author, Year, Country, N Patients	Gender, Age	Symptoms and Signs	POS (Days)	ICU	ICU LOS (Days)	Hospital LOS (Days)	Vital Status at Discharge
Caramello P., et al., 2002, Italy, 1 [28]	M (NA)	malaise, fever, headache, blurred vision, conjunctival injection, abdominal and back pain	2	no	NA	18	vital
Seitsonen E., et al., 2006, Finland, 2 [29]	M (72 years)	fever, upper abdominal pain, melena	3	yes	18	NA	vital
	M (34 years)	high fever, fatigue, myalgia, upper abdominal pain, vomiting, diarrhea	4	yes	15	18	vital
Rasmuson J., et al., 2011, Sweden, 3 [30]	F (73 years)	malaise, fever, nausea, vomiting, dyspnoea, somnolence	2	yes	13	13	deceased
	F (65 years)	fever, chills, dyspnoea, dry cough, diarrhea, syncope	4	yes	13	NA	vital
	F (63 years)	fever, chills, dyspnoea, dry cough, diarrhea	3	yes	2	NA	deceased
Gizzi M., et al., 2013, Belgium, 1 * [31]	F (42 years)	fever, thoracic pain, dyspnea, rhinorrhea, blurred vision, headache, lumbar discomfort, nausea, vomiting	4	yes	12	NA	vital
Vollmar P., et al., 2016, Germany, 1 ** [32]	F (53 years)	severe headache, radiating into the neck, chills, fatigue and vomiting	3	yes	39	55	vital
Gözdaş H.T., et al., 2018, Turkey, 1 [33]	M (24 years)	fever, fatigue, and severe headache	3	yes	1	10	vital
Sulleiro E., et al., 2020, Spain, 1 [34]	M (28 years)	fever, malaise, weakness, headache, arthralgia, abdominal pain, rash on trunk, dyspnea	2	yes	NA	7	vital

* Puumala virus, Doische, Belgium—South of Belgium, near the French border. ** The sequences were 99–100% homologous to PUUV strain Bavaria (Genbank Acc. No.: KU601446, KU601447). Lower Bavaria, South-East Germany. M = male; F = female; NA = not available; POS = post onset of symptoms; ICU = intensive care unit; LOS = length of stay.

**Table 4:** Review of cases and case series of HPS caused by PUUV published so far—intensive care medicine, immunomodulatory and antimicrobial treatment.

Author, Year, Country, N Patients	MV	VV or VA ECMO	CRRT	Vasopressor	Corticosteroids	Antivirals	Antibiotics
Caramello P., et al., 2002, Italy, 1 [28]	no	no	no	NA	NA	NA	NA
Seitsonen E., et al., 2006, Finland, 2 [29]	yes	no	yes	yes	yes	no	yes
	yes	no	yes	yes	yes	no	yes
Rasmuson J., et al., 2011, Sweden, 3 [30]	yes	no	no	yes	yes	no	yes
	yes	no	yes	yes	yes	no	yes
	yes	no	no	yes	yes	no	yes
Gizzi M., et al., 2013, Belgium, 1 [31]	no	no	yes	yes	no	no	yes
Vollmar P., et al., 2016, Germany, 1 [32]	yes	VA, VV ECMO	yes	yes	NA	Acyclovir	yes
Gözdaş H.T., et al., 2018, Turkey, 1 [33]	no	no	no	no	no	Oseltamivir	yes
Sulleiro E., et al., 2020, Spain, 1 [34]	no	no	no	yes	NA	NA	NA

NA = not available; MV = mechanical ventilation; VV ECMO = veno-venous extracorporeal membrane oxygenation; VA ECMO = veno-arterial extracorporeal membrane oxygenation; CRRT = continuous renal replacement treatment.

## Data Availability

Not applicable.
